# Too Long, Too Short, or Just Right: Glycosphingolipid–Protein Binding Varies with Acyl Chain Length

**DOI:** 10.1371/journal.pbio.0040397

**Published:** 2006-10-24

**Authors:** Richard Robinson

Glycosphingolipids (GSLs) reside in the membranes of all mammalian cells, where they play roles in both structure and signaling. They traffic between the plasma membrane—where most are found—and vesicle membranes within the cell. One of the carriers of GSLs is glycolipid transfer proteins. The interactions between these two molecules have only recently begun to be elucidated. In a new study, Lucy Malinina, Margarita Malakhova, Rhoderick Brown, Dinshaw Patel, and colleagues reveal a highly unusual binding characteristic of the protein: the sphingosine chain of the GSL either buries itself inside the protein or is left outside of it, depending on the length of the acyl chain.

Every GSL has three parts: a sugar head and two long hydrocarbon chains (an 18-carbon, nitrogen-containing sphingosine chain, and an “acyl” chain whose length can vary from 16 to 26 carbons). Using x-ray crystallography, the authors recently elucidated the structure of human glycolipid transfer protein, both with and without an attached GSL, and showed that it has a novel protein fold adapted to interacting with membranes and binding with lipids. In that study, which used a GSL containing a lactose sugar and an 18-carbon monounsaturated acyl chain, they found that the sugar binds to the exterior, while the sphingosine and acyl chains lay parallel inside a hydrophobic tunnel made from an interior fold of the protein. To explore how the protein accommodated other GSLs, they varied acyl length and sugar groups and determined the structure of these protein–GSL complexes.

To their surprise, they found that when the acyl chain was either longer (24 carbons) or shorter (8 or 12 carbons) than the one in their initial experiment, the sphingosine chain was not included in the tunnel, but instead jutted out away from the surface of the protein. While the effect on sphingosine is the same, the cause appears to be slightly different in the two cases. When the shorter acyl chain sits in the tunnel, it is joined by an extraneous free hydrocarbon, which denies sphingosine an entrance. The exact origin and role of this hydrocarbon is unknown, but it also occupies the tunnel in the unbound protein. In contrast, there is no extraneous hydrocarbon when the longer acyl chain is in the tunnel, but the chain curls around within, apparently blocking out sphingosine with its bulk. When the authors reverted to the 18-carbon acyl chain but introduced an additional chain-kinking double bond, once again sphingosine was excluded, suggesting that its ability to fit depends on both the length and shape of the acyl group. The tunnel itself expands and contracts with the changes in size of the chains within.

**Figure:**
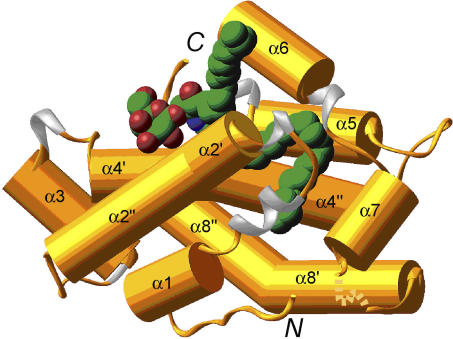
The sphingosine chain of GSL is blocked from entering the tight confines of the GLTP hydrophobic tunnel because the long acyl chain, which enters first, is forced into a serpentine-like conformation within the tunnel.

Unlike the highly variable interactions of tunnel and hydrocarbon chains, the binding of sugar to the protein appears to rely mainly on a small set of invariant attractions, whether from the double sugar, lactose, or from the single sugars, galactose or glucose. In addition, in each case there are conserved hydrogen bond contacts involving an amine and carbonyl (amide linkage) in the GSL ceramide and specific amino acids of the protein, helping to position the GSL hydrocarbons for entry into the tunnel. The binding of the amide group also triggers a conformational shift in one loop of the protein at the head of the tunnel.

From these observations, the authors propose a stepwise binding sequence for GSLs, in which the sugar binds first, acting as the primary determinant of GSL-protein specificity. The amide group binds next, orienting the GSL tails with the tunnel and shifting the loop to help open the tunnel for the hydrocarbons. The acyl hydrocarbon binds next. The sphingosine follows the acyl group into the tunnel in some cases, while in others, it remains outside.

These results should accelerate understanding of GSL trafficking within the cell and provide insight into how these molecules play their parts in cell growth and development.

